# Detection of primary Sjögren’s syndrome in primary care: developing a classification model with the use of routine healthcare data and machine learning

**DOI:** 10.1186/s12875-022-01804-w

**Published:** 2022-08-09

**Authors:** Jesper T. Dros, Isabelle Bos, Frank C. Bennis, Sytske Wiegersma, John Paget, Chiara Seghieri, Jaime Barrio Cortés, Robert A. Verheij

**Affiliations:** 1 Netherlands Institute for Health Services Research (NIVEL), Utrecht, the Netherlands; 2 National Health Care Institute, Diemen, the Netherlands; 3 Tilburg School of Social and Behavioral Sciences, Tilburg University, Tilburg, the Netherlands; 4 Faculty of Science, Computer Science, Artificial Intelligence, Vrije Universiteit, Amsterdam, the Netherlands; 5 Institute of Management, Sant ‘Anna School of Advanced Studies, Pisa, Italy; 6 Faculty of Education and Health, University Camilo José Cela, Madrid, Spain

**Keywords:** Primary Sjögren’s syndrome, Machine learning, Routine healthcare data, Primary care

## Abstract

**Background:**

Primary Sjögren’s Syndrome (pSS) is a rare autoimmune disease that is difficult to diagnose due to a variety of clinical presentations, resulting in misdiagnosis and late referral to specialists. To improve early-stage disease recognition, this study aimed to develop an algorithm to identify possible pSS patients in primary care. We built a machine learning algorithm which was based on combined healthcare data as a first step towards a clinical decision support system.

**Method:**

Routine healthcare data, consisting of primary care electronic health records (EHRs) data and hospital claims data (HCD), were linked on patient level and consisted of 1411 pSS and 929,179 non-pSS patients. Logistic regression (LR) and random forest (RF) models were used to classify patients using age, gender, diseases and symptoms, prescriptions and GP visits.

**Results:**

The LR and RF models had an AUC of 0.82 and 0.84, respectively. Many actual pSS patients were found (sensitivity LR = 72.3%, RF = 70.1%), specificity was 74.0% (LR) and 77.9% (RF) and the negative predictive value was 99.9% for both models. However, most patients classified as pSS patients did not have a diagnosis of pSS in secondary care (positive predictive value LR = 0.4%, RF = 0.5%).

**Conclusion:**

This is the first study to use machine learning to classify patients with pSS in primary care using GP EHR data. Our algorithm has the potential to support the early recognition of pSS in primary care and should be validated and optimized in clinical practice. To further enhance the algorithm in detecting pSS in primary care, we suggest it is improved by working with experienced clinicians.

**Supplementary Information:**

The online version contains supplementary material available at 10.1186/s12875-022-01804-w.

## Background

Primary Sjögren’s Syndrome (pSS) is a chronic autoimmune disease, affecting mainly salivary and lachrymal glands, primarily in post-menopausal women [1]. Due to the low prevalence, poorly understood pathophysiology and a wide variety of symptoms, pSS is under-recognized and thus heavily underdiagnosed or misclassified in clinical practice today. This has a negative impact on the time to diagnosis (TTD) [2], which typically takes place in specialized secondary care. The prevalence of pSS varies greatly across studies, with a point estimate of 0.61‰, but ranging from 0.11–37.9‰ [3]. Currently, no separate code for pSS is present in the International Classification of Primary Care (ICPC) coding system [4]. With the growing usage of real-world data derived from administrative and clinical data, new possibilities for the earlier recognition and diagnosis of complex diseases with low prevalence like pSS arise. Routinely collected healthcare data can offer additional sources of specific data collection for clinical research [5] regarding pSS and machine learning models have been shown to be useful methods to extract previously unknown and potentially useful information regarding complex diseases like pSS [6]. In this study, we aimed to apply machine learning (ML) techniques to data from routinely recorded electronic health records (EHRs) from general practitioners (GPs) and to develop an algorithm that identifies possible pSS patients at an early stage. The algorithm might be able to facilitate the diagnostic process by GPs as a clinical decision support system (CDSS) [7].

Detecting pSS in primary care can be improved, as patients with pSS are dealing with a significant delay in their diagnosis. We attempted to build a classification model which aims to predict a Diagnosis Related Group (DRG) for pSS, based on primary care data like drug prescriptions, number of visits to the GP and registered symptoms or diagnoses. Besides primary care data, patient characteristics like age and gender were also included in the model. Machine learning was used to build this model as ML can be used to deal with large amounts of data needed to train accurate classification models in complex diseases like pSS. Data sharing initiatives for pSS research have been started [8], but so far no study has been conducted using ML techniques to classify patients with pSS in primary care. This study will be the first to look at the potential of ML to classify patients with pSS in primary care, based on GP EHR data.

The aim of the study was to develop a classification model to identify possible pSS patients at an early stage. The model might be able to expedite specialist referral by GPs, by forming the basis for the development of a CDSS for GPs, as was recently been done for several other low prevalent diseases [7, 9]. Since ML models differ in terms of transparency and interpretability, both logistic regression and random forest were used and compared. A logistic regression model is more transparent and interpretable, but can only capture linear relationships between features and outcomes. A random forest model is less transparent and interpretable, but is better at handling non-linear relationships. This can be an advantage in complex and multi-dimensional datasets.

## Method

### Databases

For this study, routine healthcare data from two databases was used. Routinely recorded primary care data was obtained from the Nivel Primary Care Database (Nivel PCD). Nivel PCD contains primary care data from 10% of the Dutch population (1.7 million unique patients), enlisted in approximately 500 GP practices. Secondary care data was available from the Diagnosis Related Groups Information System (DIS), which contains routinely recorded hospital claims data (HCD) for all hospitals in the Netherlands. The DIS database contained data for 12,991,265 unique patients for the period 2012–2017.

### Patient sample

Primary care data from Nivel PCD was linked to the secondary care DIS dataset using pseudonyms of patients’ national personal identification number (PIN). The PIN is a unique, personal number and is registered for all patients by their health care providers. The pseudonymization and data handling process in Nivel Primary care database is described elsewhere [10]. Data of all individuals enlisted as patients in general practices participating in Nivel PCD for the complete period of 2012–2016 was uploaded to Statistics Netherlands for linkage to the DIS database, which is structured following the diagnosis-related group (DRG) system [11]. DRGs describe the type of care for specific diseases for each medical department. As patients with pSS are treated under three different medical specialties, we included DRGs from: Rheumatology (code 324–0308), Internal Medicine (code 313–0524) and Ophthalmology (code 301–0404). For Ophthalmology, the DRG is not specific to pSS but could also include patients with sicca syndrome. However, given the notable overlap between sicca and pSS [3], we included all patients with the 301–0404 DRG in Ophthalmology. We only included patients with an initial pSS diagnoses from 2017. We removed patients who were diagnosed before 2017. Explicitly, pSS patients in this study are defined as patients having an initial pSS diagnosis in the DIS database.

### Available primary and secondary care data

#### Feature extraction

Both symptoms and diseases are coded by GPs using the International Classification of Primary Care, version 1 [12]. For drug prescriptions, the international Anatomical Therapeutic Chemical (ATC) classification system for drugs is used on ATC-3 level [13]. For all GP contacts, reimbursable care activities are specified using predetermined codes [14]. An extensive description of these features can be found in Appendix I. Completeness of primary care EHRs is monitored, and only data from practices living up to data quality standard were included in this study, as is described in Verheij et al. (2018) [15, 16]. The main diagnoses in secondary care are specified by ICD-10 [17]. Hospital care trajectories are coded using the DRG classification system for hospital claims data [11].

Potential features for the classification model were extracted from the EHR. For patient characteristics, age and gender were extracted. In addition, ICPC codes were extracted for each patient contact, representing symptoms and diagnoses for all patients [12]. Episodes of care were calculated using the algorithms developed by Nielen et al. (2019) [18]. Prescriptions were included through the ATC on ATC-3 level, rendering a total of 267 unique prescriptions. Regarding the care activities according to the CTG-codes, we only cosidered the following consult types: consult, consult > 20 min, consult < 20 min, home visit < 20 min, home visit > 20 min, consult by phone and repeat prescriptions. Adding all these features together, we ended up with 963 potential features for the outcome of pSS in secondary care. Since only complete cases were included in the EHR and claims data, no missing cases were present.

### Data preparation

All numeric features were scaled by subtracting the mean and dividing by the standard deviation. Dichotomous and numerical features were represented in a matrix, indexing rows by patient ID. To reduce the amount of features in the dataset, features that were present in less than 5% of the cases were removed [19]. All data analyses were performed in R studio version 1.1.463.

### Data analysis

#### Exploratory data analysis

**Fig. 1:**
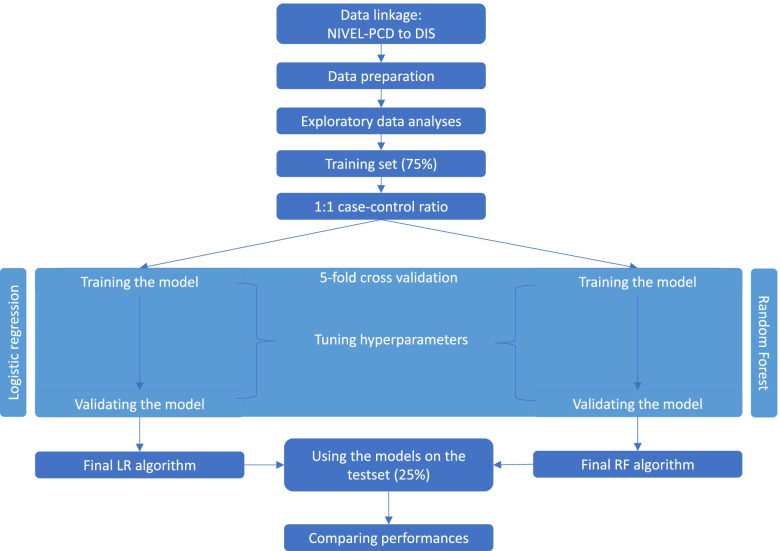
Schematic overview of the data analysis process

Figure 1 provides a schematic overview of the data analysis process. First, characteristics of the cohort were described for age and gender. We then ranked ICPC and ATC codes according to the frequency of occurrence in the different groups. Table 1 shows the 10 most frequent ICPC and ATC codes for pSS and non-pSS patients. Following these steps, the dataset was divided into a training set (75%) and a test set (25%).

**Table 1:** Patient characteristics, top 10 symptoms & diseases and top 10 prescriptions of the cohort from 2013 to 2016

	**Cohort 2013–2016 (*N*** **= 930,590)**					
	**Non-pSS (*N*** **= 929,179)**			**pSS (*N* = 1411)**		
Female (%)	487,657 (52.5)			1014 (71.9)		
Mean age female ± SD	45.9 ± 22.3			60.5 ± 16.9		
Mean age male ± SD	45.0 ± 22.3			64.0 ± 15.9		
**ICPC top 10**	**Female non-pSS**		**Male non-pSS**	**Female pSS**		**Male pSS**
	*< 45 years (N = 219,619)*	*> 45 years (N = 268,038)*	*All ages (N = 441,522)*	*< 45 years (N = 182)*	*> 45 years (N = 832)*	*All ages (N = 397)*
1.	General weakness (A04)	No disease (A97)	No disease (A97)	General weakness (A04)	General weakness (A04)	No disease (A97)
2.	No disease (A97)	High blood pressure (K86)	High blood pressure (K86)	No disease (A97)	No disease (A97)	General weakness (A04)
3.	Constipation (D12)	General weakness (A04)	General weakness (A04)	Stomach pain (D02)	Other general disease (A99)	Other general diseases (A99)
4.	Generalized abd. Pain (D01)	Other general disease (A99)	Other general disease (A99)	Allergy (A12)	Allergy (A12)	Abnormal results (A91)
5.	Allergy (A12)	Abnormal results (A91)	Excessive ear wax (H81)	Other local abd. Pain (D06)	High blood pressure (K86)	Stomach pain (D02)
6.	Fever (A03)	Stomach pain (D02)	Allergy (A12)	Other general sympt. (A29)	Stomach pain (D02)	Constipation (D12)
7.	Refractive errors (F91)	Other general sympt. (A29)	Abnormal results (A91)	Constipation (D12)	Abnormal results (A91)	High blood pressure (K86)
8.	Other general disease (A99)	Excessive ear wax (H81)	Stomach pain (D02)	Accident/injury (A80)	Adverse effect meds (A85)	Other local abd. Pain (D06)
9.	Other local abd. Pain (D06)	Allergy (A12)	Refractive errors (F91)	Iron deficiency anemia (B80)	Other general sympt. (A29)	Abnormal sensations eye (F13)
10.	Upper resp. infection (R74)	Other local abd. Pain (D06)	Asthma (R96)	Irritable bowel syndr. (D93)	Other local abd. Pain (D06)	Allergy (A12)
**ATC-3 top 10**	**Female non-pSS**		**Male non-pSS**	**Female pSS**		**Male pSS**
	*< 45 years (N = 219,619)*	*> 45 years (N = 268,038)*	*All ages (N = 441,522)*	*< 45 years (N = 182)*	*> 45 years (N = 832)*	*All ages (N = 397)*
1.	Hormonal contraceptives (G03A)	Peptic ulcer and GORD (A02B)	Lipid modifying agents (C10A)	Hormonal contraceptives (G03A)	Peptic ulcer and GORD (A02B)	Peptic ulcer and GORD (A02B)
2.	Antidepressants (N06A)	Lipid modifying agents (C10A)	Antithrombotic agents (B01A)	Antidepressants (N06A)	Lipid modifying agents (C10A)	Heparin group (B01A)
3.	Antihistamines (R06A)	Beta blocking agents (C07A)	Peptic ulcer and GORD (A02B)	Peptic ulcer and GORD (A02B)	Antithrombotic agents (B01A)	Lipid modifying agents (C10A)
4.	Anti-inflammatory and anti-rheumatic products, NS (M01A)	Antithrombotic agents (B01A)	Beta blocking agents (C07A)	Anti-inflammatory and anti-rheumatic products, NS (M01A)	Other ophthalmologicals (S01X)	Beta blocking agents (C07A)
5.	Decongestants and other nasal preparations for topical use (R01A)	Antidepressants (N06A)	Blood glucose lowering drugs, excl insulins (A10B)	Other ophthalmologicals (S01X)	Beta blocking agents (C07A)	ACE inhibitors, plain (C09A)
6.	Corticosteroids, plain (D07A)	Blood glucose lowering drugs, excl insulins (A10B)	ACE inhibitors, plain (C09A)	Decongestants and other nasal preparations for topical use (R01A)	Antidepressants (N06A)	Other ophthalmologicals (S01X)
7.	Adrenergics, inhalants (R03A)	ACE inhibitors, plain (C09A)	Antidepressants (N06A)	Anxyiolytics (N05B)	Hypnotics and sedatives (N05C)	Drugs used in benign prostatic hypertrophy (G04C)
8.	Beta-lactam antibacterials (J01C)	Anxyiolytics (N05B)	Anti-inflammatory and anti-rheumatic products, NS (M01A)	Antihistamines (R06A)	Blood glucose lowering drugs, excl insulins (A10B)	Blood glucose lowering drugs, excl insulins (A10B)
9.	Drugs for constipation (A06A)	Anti-inflammatory and anti-rheumatic products, NS (M01A)	Adrenergics, inhalants (R03A)	Opioids (N02A)	Opioids (N02A)	Selective calcium channel blockers, vascular (C08C)
10.	Peptic ulcer and GORD (A02B)	Hypnotics and sedatives (N05C)	Selective calcium channel blockers, vascular (C08C)	Corticosteroids, plain (D07A)	Calcium (A12A)	Anti-inflammatory and anti-rheumatic products, NS (M01A)

#### Training and validating the algorithms

For the training set, a case-control ratio of 1:1 was established, enabling one to train the algorithm without being influenced by the low prevalence of pSS. The training set therefore contained pSS patients and non-pSS patients. The non-pSS patients were randomly selected with the *createDataPartition* function from the caret package. The training set was used to train a logistic regression model and a random forest, with 5-fold cross validation. Both models were made using the *train* function from the R caret package. For the test set, the initial case-control ratio in the original data was kept the same. The model could thereby be tested with real-world prevalence.

#### Logistic regression

Logistic regression included a feature selection step (backwards feature selection based on the Akaike Information Criteria (AIC)). Lower AIC indicates a better model fit, and a delta-AIC (difference between two AIC values of models being compared) more than − 2 was considered significantly better [20]. Afterwards, the selected features were used for model training. The trained model was then used to find the optimal cut-off for classifying patients as non-pSS or pSS patients by predicting the outcome in the validation set with different cut-off points varying between 0 and 1. The optimal cut-off point was chosen as the point where sensitivity was equal to the specificity. This was a pragmatic choice, as the preference from GPs for either higher sensitivity or specificity is unknown. The cut-off was chosen as a neutral value which can be altered in future studies when GP preferences are known. After including all relevant features and tuning the cut-off point, the test set was used to obtain generalization to an unseen dataset. The prevalence of pSS in the validation and test set was equal to the real-world prevalence.

#### Random forest

The random forest was trained based on all features, without an extra feature selection step other than using the variables with at least 5% of all values filled as is described in the data preparation step. The number of decision trees was set at 1000. The chosen features per tree were random, which is the default. Finally, the optimal number of features per tree was optimized by choosing the number of features at which the OOB error rate does not improve any further by adding more features. Similarly to the logistic regression, the optimal cut-off point was chosen. After including all relevant features and tuning the hyper parameters, the test set was used to obtain generalization to an unseen dataset.

## Results

A total of 930,590 patients from the Nivel PCD were linked to 12,991,265 secondary care patients in the DIS database. All 930,590 patients present in the Nivel PCD could be linked to the DIS database, so combined primary and secondary care trajectories could be analyzed for these patients. We removed 28,675 pSS patients, as they were diagnosed before 2017. Ultimately, 1411 pSS patients were identified in the DIS database in 2017.

In Table 1, patient characteristics and most common prescriptions, diseases and symptoms are shown and stratified by gender and age group. For women, these descriptive statistics are also stratified by age below or above 45 years old, to check for pre- and postmenopausal differences. The chosen age of 45 was conservatively based on the age range at which the menopause usually starts [21]. On average, pSS patients were between 60.5 and 64.0 depending on gender. Most of patients were women (71.9%).

### Logistic regression

The training model contained 182 variables after removing features that were present in less than 5% of the cases. The most important features of the logistic regression (LR) model can be found in Appendix II. The 10 most important features can be found in Table 2. The optimal cut-off point was found to be 0.48. The confusion matrix of the LR model can be seen in Table 3.

**Table 2:** Feature importance for both logistic regression and random forest. GP = general practitioner, NS = non-steroid

	Logistic Regression	Random Forest
1.	S01X (other ophthalmologicals)	Age
2.	Age	S01X (other ophthalmologicals)
3.	Gender	Number of GP consults < 20 min
4.	S01A (anti-infectives for ophthalmological use)	Number of GP consults > 20 min
5.	Number of GP consults > 20 min	Number of GP consults by phone
6.	S01G (decongestants and anti-allergics for ophthalmological use)	A02B (drugs for peptic ulcer and gastro-oesopheagel reflux disease)
7.	Number of GP visitations at home < 20 min	Gender
8.	S01C (anti-inflammatory agents and anti-infectives in combination)	Repeat prescription
9.	J01F (macrolides, lincosamides and streptogramins)	N02A (opioids)
10.	A99 (other generalized/non-specified diseases)	M01A (Anti-inflammatory and anti-rheumatic products, NS)

**Table 3:** Confusion matrix for the classification of primary Sjögren Syndrome using logistic regression. pSS = primary Sjögren Syndrome, Tp = true positive, Fp = false positive, Tn = true negative, Fn = false negative

		**Secondary care**		
		**Non-pSS**	**pSS**	**Total**
Primary Care	**Non-pSS**	85,895 [Tn]	49 [Fn]	85,944
	**pSS**	30,251 [Fp]	128 [Tn]	30,379
	**Total**	116,146	177	116,323

These classifications lead to a sensitivity of 72.3%, specificity of 74.0%, negative predictive value of 99.9% and a positive predictive value of 0.4%.

### Random forest

After tuning the RF model, 18 features per decision tree was chosen as optimal, since it had the lowest OOB error rate when classifying pSS patients.. The optimal cut-off point was found to be 0.55. The confusion table of the RF is depicted in Table 4.

**Table 4:** Confusion matrix for the classification of primary Sjögren Syndrome using random forest. pSS = primary Sjögren Syndrome, Tp = true positive, Fp = false positive, Tn = true negative, Fn = false negative

		**Secondary care**		
		**Non-pSS**	**pSS**	**Total**
Primary Care	**Non-pSS**	90,513 [Tn]	53 [Fn]	90,566
	**pSS**	25,633 [Fp]	124 [Tn]	25,757
	**Total**	116,146	177	116,323

These classifications lead to a sensitivity of 70.1%, specificity of 77.9%, negative predictive value of 99.9% and a positive predictive value of 0.5%. The top 10 most important features can be found in Table 2, ranked by feature importance.

### Model overview

**Fig. 2:**
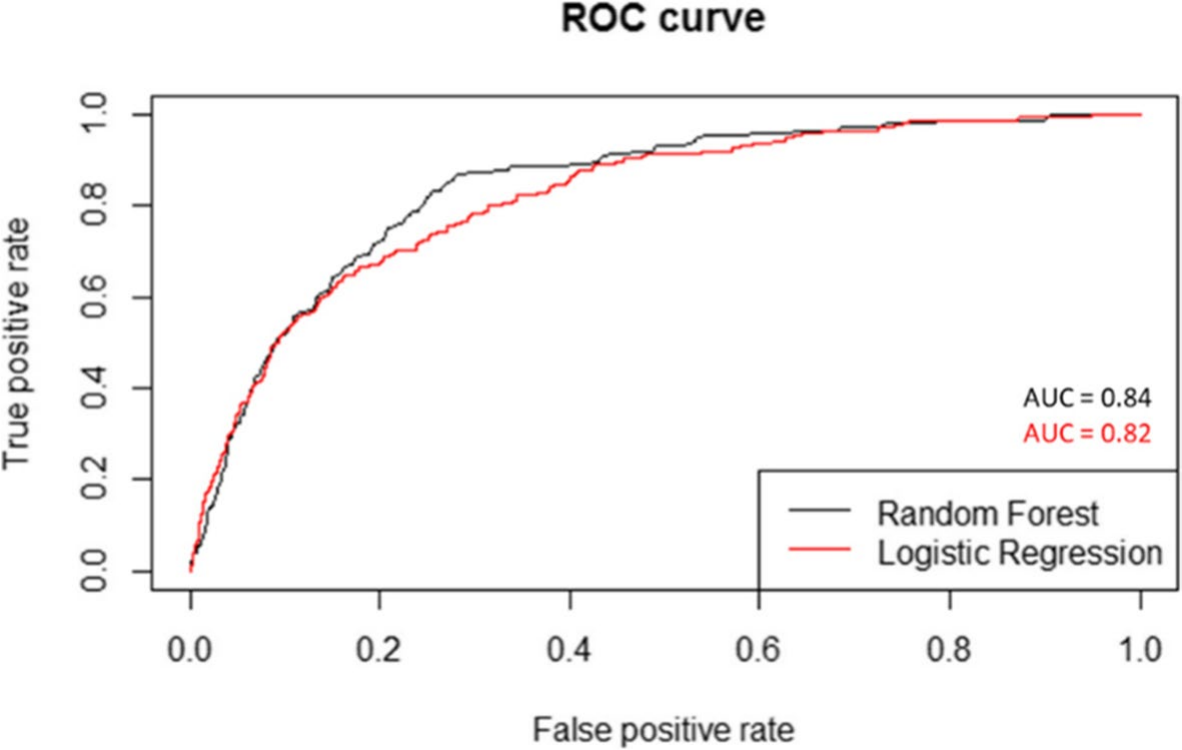
Receiver Operating Characteristic of both the logistic regression and random forest

**Fig. 3:**
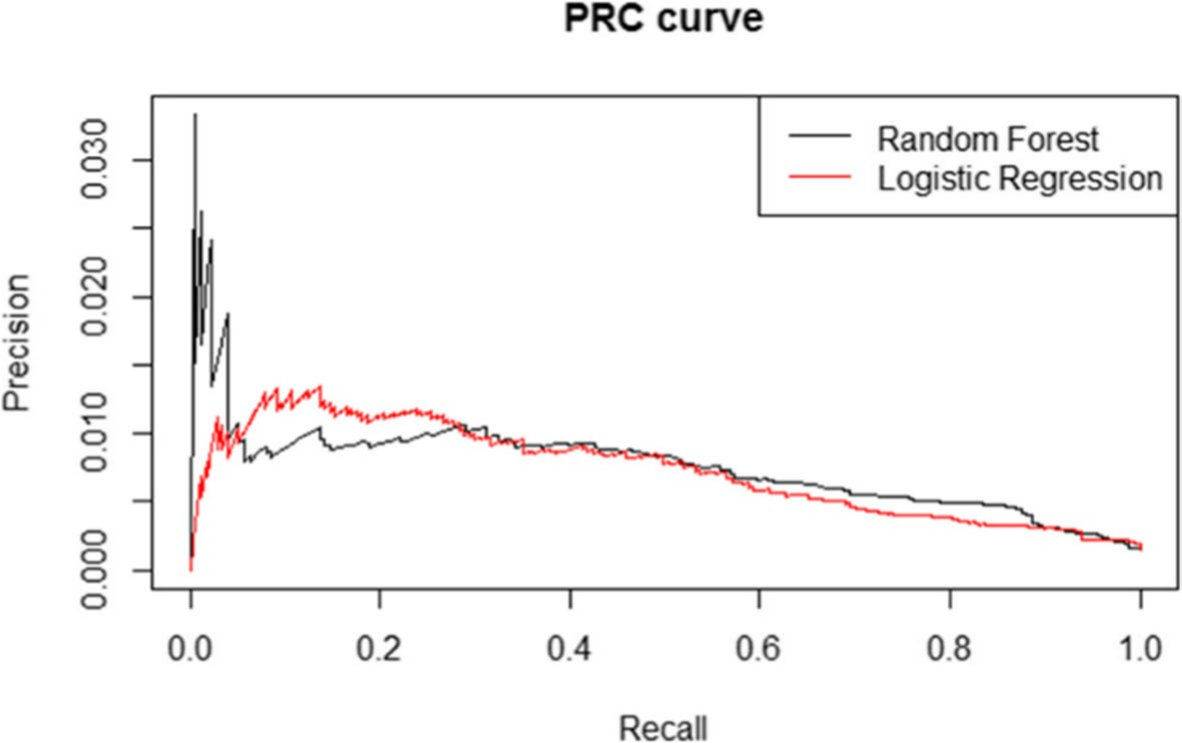
Precision Recall Curve of both the logistic regression and random forest

For both models, a Receiver Operating Characteristic (ROC, Fig. 2) and Precision Recall Curve (PRC, Fig. 3) were drawn to get a model overview for both classification models. The Area under the ROC curve (AUC) for the logistic regression model was 0.82, where the AUC for the random forest was 0.84. The area under the PR curve was 0.01 for both models.

## Discussion

The aim of this study was to develop an algorithm to detect pSS patients in primary care. This could be used as input to develop a CDSS for GPs to improve the early detection of pSS. This is the first study that looks at the potential of using machine learning to classify patients with pSS in primary care using EHR data. By linking primary and secondary care data for pSS and non-pSS patients, we were able to confirm basic but important differences between these groups reported in the literature [2, 3]. Overall, our two models performed quite well in detecting patients with pSS. However, due to the low prevalence of the disease, many false positives occurred. Both models were near perfect in classifying non-pSS patients, with 99.9% of classified non-pSS not having a pSS diagnosis. These models could be used as input to develop a CDSS in the future, but still need improvement before being useful in general practices.

Although the negative predictive value, sensitivity and specificity varied between moderately good to near perfect for both models, the positive predictive value was poor. The low positive predictive value could partly be attributed to the low prevalence of pSS, making the chance of having pSS within a population very low in the first place. The prevalence in our dataset (0.15‰) is comparable to the prevalence reported in international literature, with a range of 0.11–37.9‰ and a point estimate of 0.61‰ [3]. A study performed by Lutgendorf et al. (2016), showed that even with a sensitivity and specificity of 99.9%, the PPV was only 45% in a population with a prevalence of 0.8‰. However, with a prevalence of 5%, the PPV with the same specificity and sensitivity was almost 100%. This phenomenon is known as the false positive paradox [22, 23]. The target population when testing for a rare disease is crucial for the model performance and usefulness and is also of paramount importance for both our models. This means that positive results in a general practice setting should be interpreted with caution due to the low prevalence. Negative results, however, can be reassuring, as the negative predictive value has a very low error rate.

Key features of the models were S01X (other ophthalmologicals), N02A (opioids), F13 (abnormal sensations in eye), age, gender and number of visits to the GP. These features are in line with reported symptoms in the literature [1–3, 24]. However, L99 (other musculoskeletal diseases), F99 (other diseases eyes) and non-Hodgkin’s disease (B72.02) were not as important as expected when compared to previous studies [4, 24]. None of the three diseases above were in the feature importance top-15 for either of the models. Of these features, F99 was the most important feature, ranked 23th in the RF and 42th in the LR. The RF had a lower sensitivity and a better specificity compared to the LR model, but even though feature importance can be calculated, the exact build-up of the final RF model is difficult to interpret. This is due to the characteristic ‘black box’ for which RF is known [25], whereas the LR model is more transparent and understandable. In practice, interpretation of the LR model might be of high value for clinicians, even though this could mean a slight underperformance compared to the RF. Also, even though model performance of the RF was stable during the cross validation procedure, it was more prone to overfitting (as can be seen in Appendix III) and the LR model might be a more safe model when used on an external dataset.

This study has several limitations. First, the data was derived from primary care EHRs and medical claims data from secondary care, for which no data is known before 2013 in case of the latter. It could be that some of the patients were diagnosed with pSS before 2013 in secondary care but did not have any medical claims after 2013 and we have missed these patients (and are therefore considered to be non-pSS patients). Second, the diagnosis of pSS in the medical claims database was the outcome feature in this study, for which the reliability is unknown. However, no potential bias (i.e., over- or underestimation of the actual amount of pSS patients) is expected, as there is no known incentive for either over- or underdiagnosing patients with pSS in secondary care. As stated in the method section, no discrimination between pSS and sicca syndrome is made within the ophthalmologic DRG for pSS. Therefore, part of the outcome feature might be sicca syndrome and not pSS, as we did not want to exclude any potential pSS patients. Third, the quality of the determinants on which the classification models is trained, is of high importance as well [26]. These determinants are derived from EHRs, which carries a risk for error. Because even though EHR data will probably be more reliable than claims data, as its purpose is to facilitate the medical process, its purpose is not to provide information for clinical research. Finally, different doctors may use different classification criteria to diagnose pSS, as is reported by Argyropoulou et al. (2018) [27]. The strengths of this study are the inclusion of real-world data from many patients with an until now relatively poor understood illness, the long period over which the data has been analyzed and a unique combination of primary and secondary care data for patients with pSS.

To our knowledge, this is the first study which looks at the potential of using machine learning to classify patients with pSS in primary care using EHR data from the GP. Previous machine learning studies in pSS have focused on 1) determinants of diagnoses based on hospital EHR data [28], 2) pathogenesis based treatments [29] or 3) identifying pSS subtypes [6]. This study resembles the first exploration of the potential of machine learning methods for classifying patients with pSS in primary care. Since the purpose of data does not always fit with the use of this data in classification and prediction models, external validation of these models is extremely important to confirm their performance in the real world. Throughout the literature, studies on prediction and classification models are notorious for their lack of (external) validation [30]. A systematic review comparing ML with health professional assessment in diagnosis of various diseases from medical images showed that only 24% of the studies evaluated the performance of their algorithm on external data [26]. Moreover, only 17% compared this out-of-sample performance of their algorithm with the assessment of actual health professionals. Adding to this is the point that low prevalence diseases like pSS are even more complex to assess, and this makes the results of a recent scoping review on the use of ML in rare diseases highly relevant. This study found that only 11.8% of current studies validated their classification models on an external data set and even fewer on a human expert (2.4%) [31]. Our models should therefore be validated in the future with the use of external datasets. Including frontline clinicians in this process will be critical to further evaluate the algorithm and to further advance success in the field of early detection of pSS in primary care.

## Conclusion

Our study shows that machine learning techniques can classify patients as having pSS with the use of routine healthcare data. These results can be used to further develop a CDSS for detecting pSS in primary care. Combining big data with machine learning techniques is a promising approach, but careful considerations for the selection and interpretation of real-world data are needed. Future studies should validate these models based on external datasets and in real-world settings, in order to bridge the gap between research and clinical practice.

## Supplementary Information

**Additional file 1: Appendix I.** Feature description. An extensive description of features used during our study.

**Additional file 2: Appendix II.** Used features and their importance. Feature importance (model explainability analysis) that shed light into the most important features for classifying patients with primary Sjogren Syndrome.

**Additional file 3: Appendix III.** ROC curves on training and testing data. plots for the train and validation loss.

## Data Availability

The data that support the findings of this study are available from Nivel but restrictions apply to the availability of these data, which were used under license for the current study, and so are not publicly available. Data are however available from the authors upon reasonable request and with permission of Nivel.

## Abbreviations

AIC: Akaike Information Criteria
ATC: Anatomical Therapeutic Chemical
CDSS: Clinical Decision Support System
DIS: Diagnosis Related Groups Information System
DRG: Diagnosis Related Group
EHR: Electronic Health Records
GP: General Practitioner
HCD: Hospital Claims Data
ICPC: International Classification of Primary Care
LR: Logistic Regression
ML: Machine Learning
OOB: Out-Of-Bag
PIN: Personal Identification Number
PRC: Precision Recall Curve
PSS: Primary Sjögren’s Syndrome
RF: Random Forest
ROC: Receiver Operating Characteristic
TTD: Time To Diagnosis
