# Supplementary material for: Detection of primary Sjögren’s syndrome in primary care: developing a classification model with the use of routine healthcare data and machine learning
Source: BMC Prim Care. 2022 Aug 9;23:199. doi: 10.1186/s12875-022-01804-w (PMC9361661; doi:10.1186/s12875-022-01804-w)
Supplement: Supplementary file 1 — Additional file 1: Appendix I. Feature description. An extensive description of features used during our study. [file 12875_2022_1804_MOESM1_ESM.docx]

**Appendix I: feature description**

### **Patient and practice characteristics**

Extracted patient features were gender (1 = male, 2 = female) and age. Practice ID and patient ID were nominal features containing a pseudonym of the actual ID.

### **Diseases and symptoms**

In primary care, diagnoses are recorded according to the International Classification of Primary Care (ICPC, version 1). This classification system clusters diseases in 17 chapters (e.g., Blood (B), Neurological (N), Respiratory (R)), and each chapter covers both symptoms (codes 1-29) and diseases (codes 70-99). The codes can be further divided into main codes (the chapter letter followed by a two-digit number) and sub codes. For example, for the main code T06 (Anorexia nervosa/bulimia), the sub codes are T06.01 (Anorexia) and T06.2 (Bulimia). Diseases and symptoms features were extracted at the main chapter and the main code level T06.

### **Prescriptions**

Drug prescriptions are recorded using the Anatomical Therapeutic Chemical (ATC) classification system for medicines. This system clusters drugs in groups based on the organ or system in which they act, therapeutic intent, and chemical characteristics (ref). The ATC system clusters drugs at five levels. The first level divides drugs into 14 anatomical groups, the second level adds the therapeutic subgroup, the third level adds the pharmacological subgroup, the fourth adds the chemical subgroup, and the fifth the chemical substance. Drug prescriptions were extracted at ATC level 3, so at therapeutic/pharmacological subgroup level, which are in the format “*C03C High-ceiling diuretics*”.

### **Health services**

Health services are coded using the Dutch CTG-codes for medical activities conducted at the general practice. Due to data upload limitations, we only included consult types (12000-12005).
