# Supplementary material for: Detection of primary Sjögren’s syndrome in primary care: developing a classification model with the use of routine healthcare data and machine learning
Source: BMC Prim Care. 2022 Aug 9;23:199. doi: 10.1186/s12875-022-01804-w (PMC9361661; doi:10.1186/s12875-022-01804-w)
Supplement: Supplementary file 2 — Additional file 2: Appendix II. Used features and their importance. Feature importance (model explainability analysis) that shed light into the most important features for classifying patients with primary Sjogren Syndrome. [file 12875_2022_1804_MOESM2_ESM.docx]

# **Appendix II: used features and their importance**

| Variable | Importance |
| --- | --- |
| S01X (Other ophthalmologicals) | 9,74487472 |
| age | 8,600807059 |
| gender | 6,101037551 |
| S01A (anti-infectives for ophthalmological use) | 4,14983396 |
| `12001` (Number of GP consults >20 min) | 3,781506201 |
| S01G (decongestants and anti-allergics for ophthalmological use) | 3,456399193 |
| `12002` (Number of GP visitations at home <20 min) | 3,430329762 |
| S01C (anti-inflammatory agents and anti-infectives in combination) | 3,064973257 |
| J01F (macrolides, lincosamides and streptogramins) | 2,917527173 |
| `1099` (other generalized/non-specified diseases) | 2,913582769 |
| `1692` (shoulder syndrome) | 2,894752961 |
| `1391` (Refractive errors) | 2,818948119 |
| `1701` (Headache (excl. N02 N89 R09)) | 2,791427016 |
| `2012` (Insect bite) | 2,709641063 |
| C09D (ANGIOTENSIN II RECEPTOR BLOCKERS (ARBs), COMBINATIONS) | 2,606539088 |
| A11C (VITAMIN A AND D, INCL. COMBINATIONS OF THE TWO) | 2,587052263 |
| A12A (Calcium supplements) | 2,477207614 |
| `1689` (Osteoarthritis of hip) | 2,475842871 |
| `1399` (Other disease of eye) | 2,475326631 |
| A03F (PROPULSIVES) | 2,471703352 |
| `1695` (Osteoporosis) | 2,349726474 |
| R05D (COUGH SUPPRESSANTS, EXCL. COMBINATIONS WITH EXPECTORANTS) | 2,318078008 |
| `12005` (repeat prescription) | 2,306801937 |
| M01A (Anti-inflammatory and anti-rheumatic products, NS) | 2,11917131 |
| A02B (Peptic ulcer and gastro-esophageal reflux disease) | 2,087947701 |
| `1220` (Sympt/complt mouth/tongue/lip) | 2,010655283 |
| `1273` (Presumed GastroIntestinal infection) | 1,934810174 |
| N03A (ANTIEPILEPTICS) | 1,906063449 |
| `1595` (Varicose veins of legs (excl S97)) | 1,836552691 |
| G04B (UROLOGICALS) | 1,773812495 |
| `2079` (Other benign neoplasm of skin) | 1,757701997 |
| C05A (TREATMENT OF HEMORRHOIDS AND ANAL FISSURES FOR TOPICAL USE) | 1,75537657 |
| `1602` (Back symptoms/complaints) | 1,750647369 |
| `1902` (Shortness of breath dyspnea) | 1,711971263 |
| H03A (THYROID PREPARATIONS) | 1,679616645 |
| C07A (Beta blocking agents) | 1,657583834 |
| `2411` (Menopausal sympt/complt) | 1,655262867 |
| `1305` (Oth visual sympt/compl (excl F94)) | 1,655084133 |
| `1995` (Emphysema/COPD) | 1,617437957 |
| `2193` (Lipid metabolism disorder) | 1,614341751 |
| D06A (ANTIBIOTICS FOR TOPICAL USE) | 1,502328558 |
| `1202` (Stomach pain/ache) | 1,49884129 |
| `1507` (Swollen ankles/edema) | 1,483681956 |
| `1684` (Osteoarthritis of spine) | 1,478586704 |
| `1688` (Rheumatoid arthrit/allied cond) | 1,478461212 |
| `1975` (Sinusitis acute/chron) | 1,470681313 |
| `1004` (General weakness/tiredness) | 1,440910117 |

Supplemental Table 1: variable importance top 50 for the logistic regression model.

| Variable | IMPORTANCE |
| --- | --- |
| age | 75,15315923 |
| S01X (Other ophthalmologicals) | 66,5854581 |
| `12000` (Number of GP consults <20 min) | 49,46220084 |
| `12001` (Number of GP consults >20 min) | 48,06809397 |
| `12004` (Number of GP consults by phone) | 42,11773437 |
| A02B (drugs for peptic ulcer and gastro-oesopheagel reflux disease) | 22,15036201 |
| gender | 15,9732853 |
| `12005` (repeat prescription) | 15,74832823 |
| N02A (opioids) | 11,50221511 |
| M01A (Anti-inflammatory and anti-rheumatic products, NS) | 11,15562405 |
| X1313 (Abnormal sensations in eye) | 10,60162098 |
| S01A (anti-infectives for ophthalmological use) | 10,2350155 |
| X1586 (Uncomplicated hypertension) | 9,693320798 |
| A11C (VITAMIN A AND D, INCL. COMBINATIONS OF THE TWO) | 9,149357045 |
| A06A (Drugs for constipation) | 8,869366551 |
| X1392 (Cataract) | 8,477590398 |
| X1399 (Other disease of eye) | 8,387579621 |
| S01C (anti-inflammatory agents and anti-infectives in combination) | 8,151397714 |
| X2193 (Lipid metabolism disorder) | 7,513681087 |
| H02A (CORTICOSTEROIDS FOR SYSTEMIC USE, PLAIN) | 7,242519691 |
| X1097 (No disease) | 7,23045658 |
| R01A (Decongestants and other nasal preparations for topical use) | 7,210199009 |
| S01B (ANTIINFLAMMATORY AGENTS) | 7,098791577 |
| A12A (Calcium) | 7,085787371 |
| D07A (CORTICOSTEROIDS, PLAIN) | 6,985424338 |
| J01C (BETA-LACTAM ANTIBACTERIALS, PENICILLINS) | 6,940477307 |
| `12002` (Number of GP visitations at home <20 min) | 6,671867019 |
| J01A (TETRACYCLINES) | 6,642640734 |
| X2099 (Other dis skin/subcut tissue) | 6,556329405 |
| B01A (ANTITHROMBOTIC AGENTS) | 6,395402938 |
| N05B (ANXIOLYTICS) | 6,373891801 |
| X1603 (Low back complt excl radiation) | 6,36037683 |
| C10A (LIPID MODIFYING AGENTS, PLAIN) | 6,218550794 |
| `12003` (Number of GP visits >20 min) | 6,213869903 |
| X1974 (Upper Resporatory Infection (head cold)) | 5,716887112 |
| X1004 (General weakness/tiredness) | 5,648624187 |
| X1905 (Cough) | 5,62288655 |
| R06A (ANTIHISTAMINES FOR SYSTEMIC USE) | 5,591135003 |
| X1602 (Back symptoms/complaints) | 5,538374262 |
| D01A (ANTIFUNGALS FOR TOPICAL USE) | 5,515795378 |
| X2271 (Cystitis/other urin infect NOS) | 5,352565968 |
| X1608 (Shoulder symptoms/complaints) | 5,302982225 |
| S01G (DECONGESTANTS AND ANTIALLERGICS) | 5,263879579 |
| X1699 (Other diseases musculoskeletal system) | 5,241350431 |
| X1595 (Varicose veins of legs (excl S97)) | 5,170555669 |
| D02A (EMOLLIENTS AND PROTECTIVES) | 5,140373702 |
| X1481 (Excessive ear wax) | 5,120197127 |

Supplemental Table 2: variable importance top 50 for the random forest model.
