# Supplementary material for: Detection of primary Sjögren’s syndrome in primary care: developing a classification model with the use of routine healthcare data and machine learning
Source: BMC Prim Care. 2022 Aug 9;23:199. doi: 10.1186/s12875-022-01804-w (PMC9361661; doi:10.1186/s12875-022-01804-w)
Supplement: Supplementary file 3 — Additional file 3: Appendix III. ROC curves on training and testing data. plots for the train and validation loss. [file 12875_2022_1804_MOESM3_ESM.docx]

# **Appendix III: ROC curves on training and testing data**


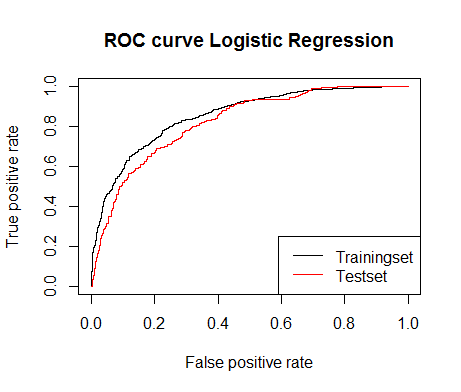


*Supplementary Figure 1: ROC curve showing the difference between LR model performance on the training and testing data.*


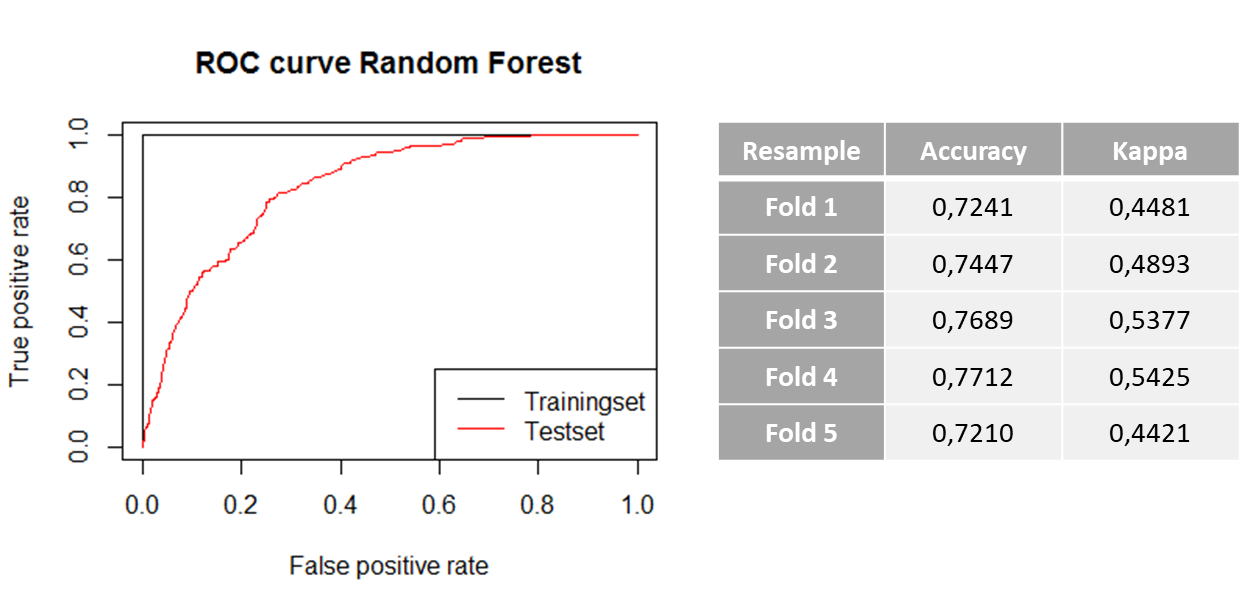


*Supplementary Figure 2: ROC curve showing the difference between RF model performance on the training and testing data. In the training data, there is a lot of overfitting going on. However, in the table shown to the right of the ROC curve, testing performance is shown of all folds during cross validation. As accuracy is relatively constant, the effect of overfitting on model performance seems to be small.*
